# Data Resource Profile: Extramural Leiden University Medical Center Academic Network (ELAN)

**DOI:** 10.1093/ije/dyae099

**Published:** 2024-07-24

**Authors:** Janet M Kist, Hedwig M M Vos, Rimke C Vos, Albert T A Mairuhu, Jeroen N Struijs, Robert R J M Vermeiren, Petra G van Peet, Hendrikus J A van Os, Frank H Ardesch, Edith D Beishuizen, Yvo W J Sijpkens, Margot W M de Waal, Marcel R Haas, Rolf H H Groenwold, Mattijs E Numans, Dennis Mook-Kanamori

**Affiliations:** Department of Public Health & Primary Care, National eHealth Living Lab and Health Campus, Leiden University Medical Center, The Hague and Leiden, The Netherlands; Department of Public Health & Primary Care, National eHealth Living Lab and Health Campus, Leiden University Medical Center, The Hague and Leiden, The Netherlands; Department of Public Health & Primary Care, National eHealth Living Lab and Health Campus, Leiden University Medical Center, The Hague and Leiden, The Netherlands; Department of Internal Medicine, HAGA Teaching Hospital, The Hague, The Netherlands; Department of Public Health & Primary Care, National eHealth Living Lab and Health Campus, Leiden University Medical Center, The Hague and Leiden, The Netherlands; Department of National Health and Healthcare, National Institute for Public Health and the Environment, Bilthoven, The Netherlands; Department of Child and Adolescent Psychiatry LUMC Curium, Leiden University Medical Centre, Leiden, The Netherlands; Parnassia Psychiatric Institute, The Hague, The Netherlands; Department of Public Health & Primary Care, National eHealth Living Lab and Health Campus, Leiden University Medical Center, The Hague and Leiden, The Netherlands; Department of Public Health & Primary Care, National eHealth Living Lab and Health Campus, Leiden University Medical Center, The Hague and Leiden, The Netherlands; Department of Public Health & Primary Care, National eHealth Living Lab and Health Campus, Leiden University Medical Center, The Hague and Leiden, The Netherlands; Department of Internal Medicine, HMC Hospital, The Hague, The Netherlands; Department of Internal Medicine, HMC Hospital, The Hague, The Netherlands; Department of Public Health & Primary Care, National eHealth Living Lab and Health Campus, Leiden University Medical Center, The Hague and Leiden, The Netherlands; Department of Public Health & Primary Care, National eHealth Living Lab and Health Campus, Leiden University Medical Center, The Hague and Leiden, The Netherlands; Department of Clinical Epidemiology, Leiden University Medical Centre, Leiden, The Netherlands; Department of Biomedical Data Science, Leiden University Medical Centre, Leiden, The Netherlands; Department of Public Health & Primary Care, National eHealth Living Lab and Health Campus, Leiden University Medical Center, The Hague and Leiden, The Netherlands; Department of Public Health & Primary Care, National eHealth Living Lab and Health Campus, Leiden University Medical Center, The Hague and Leiden, The Netherlands

Key Features

- The Extramural Leiden University Medical Center Academic Network (ELAN) dynamic data infrastructure, based on routine healthcare data of general practitioners, hospitals and mental healthcare, linked to municipal and national data sources from Statistics Netherlands, was established with the aim to facilitate broad research topics to improve integrated patient care and health outcomes.
- The dynamic data infrastructure comprises 2.6 million individuals spanning all age groups residing in South Holland province from 2007 onwards, with a diverse mix of urban and rural residents. This data infrastructure is updated on an annual basis. If residents relocate out of the area, information on death diagnoses, medication and morbidity is still accessible through national data sources.
- ELAN has coded clinical measurements, laboratory findings, morbidities, medication use, healthcare contacts and referrals. By pseudonymized linkage procedures, claims data, death diagnoses and social determinants of health are also included. Although free text is not readily available in the data set, lifestyle information data have been extracted by applying text mining and natural language processing within the separate data sources (e.g. electronic patient files).
- ELAN represents a collaborative effort of the regional academic and regional healthcare organizations.
- Requests for research collaborations and questions regarding procedures for data access, can be addressed to ELAN@LUMC.NL.

## Data resource basics

The Extramural Leiden University Medical Center Academic Network (ELAN) was developed to facilitate healthcare research concerning the major sustainable healthcare challenges Europe and the Netherlands face due to increasing healthcare costs, shortages in healthcare workers, an ageing population and persisting health disparities.^1–4^

These health disparities remain within and among European countries, with differences in healthy life years between countries reaching up to 20 years.^2^^,^^5^ The European Union considers these health disparities an important population health challenge for Europe and reported ‘a need for improved data, measurements, reporting, and comparisons; and for dedicated, collaborative research’.^6^^,^^7^

Health disparities are difficult to capture through traditional prospective cohorts because of an underrepresentation of low socio-economic groups and ethnic minorities, and due to healthy applicant bias or exclusion of patients based on (non-native) language difficulties.^8^ Also, although routine healthcare combined with national data sources have their limitations, routine healthcare and national data sources are more inclusive of patients of different socio-economic and ethnic backgrounds. Routine healthcare databases and registries are increasingly employed by healthcare organizations and governmental bodies for research and monitoring of healthcare and health policy improvement programmes.^9–12^ The Netherlands is a country with a universal healthcare system, in which all citizens are registered with a general practitioner (GP) and all registered citizens are eligible for healthcare coverage in primary and secondary care, leading to a potential integral data source of information from routine healthcare. However, routine healthcare information on individual patients in the Netherlands is dispersed in separate data information systems of municipal, primary, secondary healthcare and national data sources. Apart from linkage based on hospital and death diagnoses, these separate routine healthcare systems were previously not linked across their silo boundaries for monitoring healthcare changes or research purposes.

To study these healthcare challenges in the whole spectrum of primary and secondary routine healthcare data across the silo boundaries, ELAN was established in 2019 by the regional healthcare organizations of primary and secondary care and public health organizations.^3^ When available, data of regional routine healthcare data sources were retrospectively included from the year 2007 onwards. ELAN is funded by regional healthcare organizations, Leiden University and Leiden University Medical Centre.

**Figure 1.**
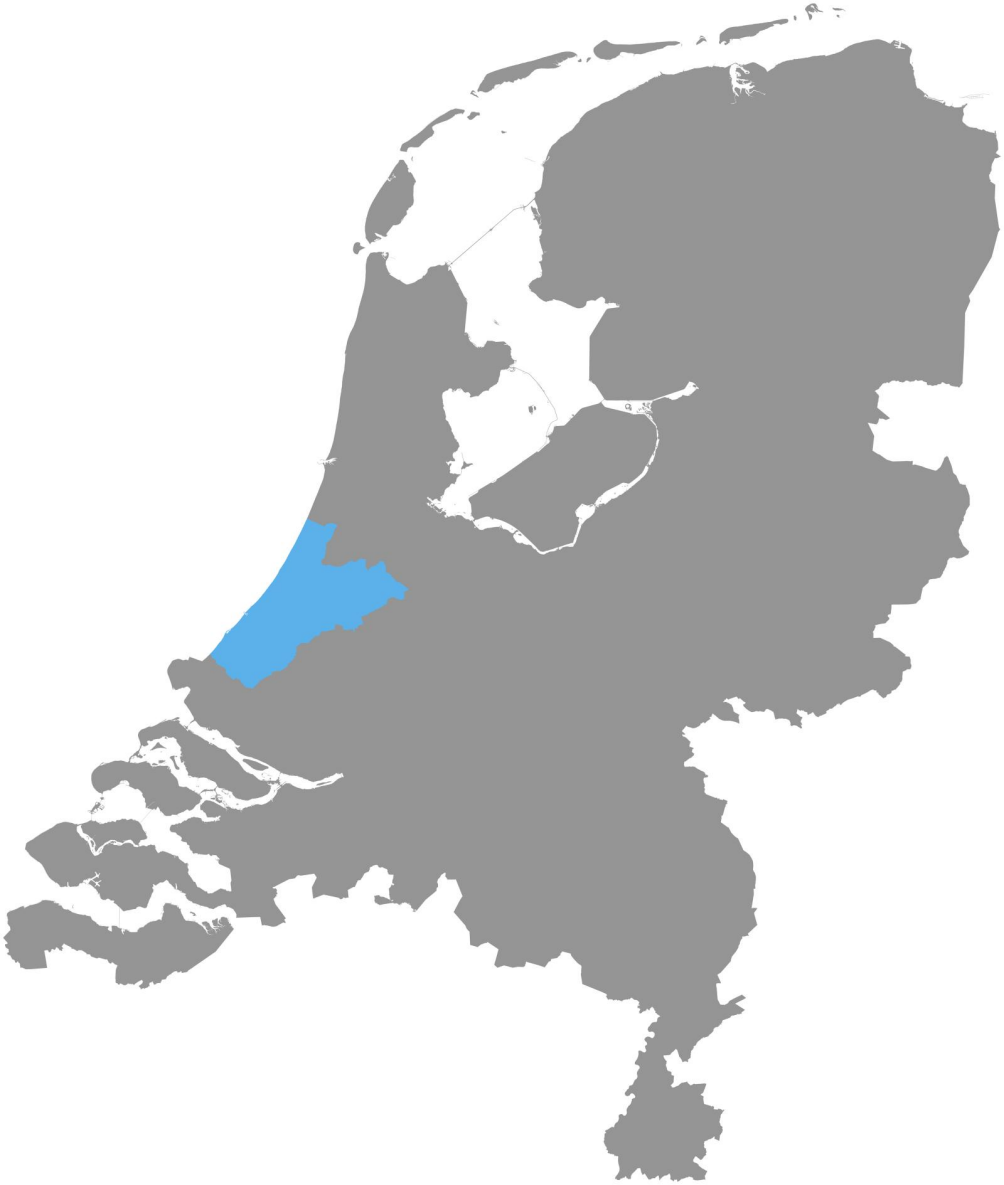
Region of the Extramural Leiden University Medical Center Academic Network (ELAN) in the Netherlands, middle and northern parts of the province of South Holland

ELAN is sourced from various data sets to form a dynamic population-based data infrastructure in the Netherlands containing information of 2.6 million individuals from the cities of The Hague, Zoetermeer, Leiden and the surrounding more rural areas (the middle and northern part of South Holland province) (Figure 1). ELAN includes regional routine healthcare information from different sources: GP practices, hospitals and mental healthcare linked to national data sources from Statistics Netherlands and information from municipalities. ELAN includes individuals from a large variety of areas, ranging from extremely urbanized areas (the cities of The Hague and Zoetermeer), suburban areas (e.g. Rijswijk) and also a rural area (e.g. the Dune and Flower bulb area). Compared with the Dutch average, the urban population is relatively oversampled within ELAN (in the ELAN area, 97% of the population live in an address-dense area compared with the Dutch average of 67%) (Table 1). The population in ELAN is more ethnically diverse than that in the Netherlands in general. In comparison with the national average, the proportion of the highest income group is larger compared with national data (quintile groups based on national disposable household income) and the proportion of the lowest income group in ELAN is smaller, except for the data from the cities of The Hague and Zoetermeer, where the lowest income group is larger (Supplementary Table S1, available as Supplementary data at *IJE* online). Regarding comorbidities, mortality by grouped major causes of death in ELAN is comparable with the Netherlands as a whole, although cardiovascular mortality and mortality due to neoplasms are somewhat lower (potentially because the population is younger) (Table 2).

**Table 1.** Characteristics of the Extramural Leiden University Medical Center Academic Network (ELAN) population, by data source, 2007–22, and the population of the Netherlands, 2014

	ELAN						
Characteristic	**SN** ^a^	**GPs** ^b^	**Hospitals** ^c^	**MHC** ^d^	**Perined children** ^e^	**Perined mothers** ^e^	**The Netherlands** ^f^
Total population^g^	2 611 953	734 519	2 168 282	409 020	371 035	239 615	16 829 289
Mid-year population 2021^h^	1 719 617	572 930	1 595 887	286 572	316 760	183 964	17 533 043
Women (%)	50.0	51.5	51.2	54.1	49.0	100.0	50.0
Age (years) [mean (SD)]	31.7 (22.6)	32.1 (23.6)	32.3 (23.0)	33.0 (20.0)	2.1 (4.1)	29.2 (7.8)	41.0
Person-years [median (IQR)]	12.4 (3.7–16.0)	16.0 (8.1–16.0)	15.4 (6.5–16.0)	16.0 (7.6–16.0)	10.7 (5.5–16.0)	16.0 (8.7–16.0)	NA
Age category (years) (%)							
<18	27.8	32.4	28.9	23.6	96.9	6.9	20.2
18–65	63.1	57.6	61.4	68.2	3.1	93.1	61.9
≥65	9.1	10.0	9.7	8.3	NA	NA	17.9
Urbanization (addresses/km^2^)^i^ (%)							
Extremely urbanized (>2500)	66.6	58.2	64.8	69.7	66.0	68.8	21.0
Strongly urbanized (1500–2500)	17.1	32.5	18.4	16.6	19.7	18.8	24.1
Moderately urbanized (1000–1500)	13.0	6.8	13.1	11.1	11.4	9.8	17.9
Hardly urbanized (500–1000)	3.3	2.3	3.6	2.7	3.0	2.6	18.5
Not urbanized (<500)	0.0	0.2	0.0	0.0	0.0	0.0	18.5
Disposable household income^j^ (%)							
1st quintile (lowest)	23.7	17.4	21.8	26.9	22.4	23.3	20.0
2nd quintile	15.6	16.4	15.7	17.4	16.2	15.2	20.0
3rd quintile	17.9	19.5	18.5	18.6	20.7	18.8	20.0
4th quintile	20.0	22.3	20.8	19.0	20.8	21.0	20.0
5th quintile (highest)	22.8	24.4	23.3	18.0	19.9	21.8	20.0
Education level^k^ (%)							
Primary to lower secondary	37.9	40.3	36.8	27.0	80.2	16.3	33.2
Higher secondary or vocational	28.6	30.3	29.4	38.5	18.0	34.0	38.6
Bachelor’s or master’s	33.5	29.4	33.8	34.4	1.8	49.7	28.2
Country of origin^l^ (%)							
The Netherlands	58.5	67.5	66.2	66.9	64.9	60.5	78.6
Surinam	3.6	3.7	4.1	5.6	3.5	5.0	2.1
Morocco	2.6	2.9	2.9	3.6	5.0	4.5	2.2
Indonesia	2.6	2.9	2.7	2.9	0.6	2.9	2.2
Middle or eastern Europe	5.6	2.7	3.3	1.8	3.0	4.0	1.2
Turkey	2.9	2.3	3.0	3.6	4.1	4.1	2.4
Antillean isles	1.8	1.7	1.8	2.0	2.0	2.1	1.0
UK	1.3	1.0	0.9	0.9	0.9	0.8	0.5
Germany	0.8	1.0	0.9	0.9	0.1	0.5	2.2
Other countries	20.2	14.3	14.2	11.9	15.9	15.8	10.9

GP, general practice; IQR, interquartile range; MHC, mental healthcare; NA, not available (unavailable or undisclosed due to low numbers); SN, Statistics Netherlands.

a Data on the citizens of the ELAN area from SN and the municipalities.

b Data on the citizens of the ELAN area from the affiliated GPs.

c Data on citizens of the ELAN area with a hospital claims declaration.

d Data on the citizens of the ELAN area with a mental healthcare claims declaration.

e Data on the citizens of the ELAN area from the Perined population (national data source with information on pregnancy and perinatal care).

f Open-source data on the population of the Netherlands from SN (year 2014 and mid-year population 2021) [13,14].

g Total population for years 2007–22.

h Population on 1 July 2021.

i Urbanization defined by number of addresses per squared kilometre.

j Quintile boundaries of disposable household income by the quintile distribution of the Netherlands.

k Highest attained and followed education level.

l Country of origin of individual or one/both parents (nine largest subgroups of the area).

**Table 2.** Mortality grouped by main causes of death in the Extramural Leiden University Medical Center Academic Network (ELAN) population by data source, 2021

	ELAN						
Mortality	**SN** ^a^	**GPs** ^b^	**Hospitals** ^c^	**MHC** ^d^	**Perined children** ^e^	**Perined mothers** ^e^	**The Netherlands** ^f^
Mid-year population	1 719 617	572 930	1 595 887	286 572	316 760	183 964	17 533 043
Causes of death (deaths/100 000 mid-year population)^g^							
Neoplasms	239	266	256	216	NA	46	268
Diseases of the circulatory system	181	174	193	177	NA	8	213
Diseases not elsewhere classified	60	51	62	71	NA	8	52
Mental and behavioural disorders	57	41	60	134	NA	NA	67
External causes of morbidity and mortality	54	52	57	103	NA	7	52
Diseases of the respiratory system	50	45	53	61	NA	NA	57
Diseases of the nervous system	40	33	43	80	NA	NA	47
Diseases of the digestive system	26	24	28	34	NA	NA	28
Endocrine and metabolic diseases	21	18	23	27	NA	NA	20
Diseases of the genitourinary system	20	19	21	21	NA	NA	20
Certain infectious and parasitic diseases	14	12	15	19	NA	NA	16
Diseases of the musculoskeletal system	6	4	7	8	NA	NA	6
Perinatal period	3	NA	1	NA	NA	NA	2
Congenital malformations	3	NA	3	NA	NA	NA	2
Diseases of the blood (-forming organs)	3	3	3	NA	NA	NA	3
Diseases of the skin	2	2	2	NA	NA	NA	2

GP, general practice; MHC, mental healthcare; NA, not available (unavailable or undisclosed due to low numbers); SN, Statistics Netherlands.

a Data on the citizens of the ELAN area from SN and the municipalities.

b Data on the citizens of the ELAN area from the affiliated general practices.

c Data on citizens of the ELAN area with a hospital claims declaration.

d Data on the citizens of the ELAN area with a mental healthcare claims declaration.

e Data on the citizens of the ELAN area from the Perined population (national data source with information on pregnancy and perinatal care).

f Open-source data on the population of the Netherlands from SN [13].

g Mortality from the Dutch National Death Registry, grouped by main causes of death according to the International Classification of Diseases, 10th revision (Grouping, Supplementary Table S2, available as Supplementary data at *IJE* online).

The participating GP practices, hospitals and other data suppliers inform their patients about pseudonymized use of their healthcare data for research purposes, and individuals can withdraw via an informed opt-out procedure. Opt-out preference is digitally marked in the records of patients, and these data are excluded from data sharing. For each new study, consent is required from the ELAN research board and from the data suppliers (e.g. GP network, hospitals, municipality, mental healthcare, Statistics Netherlands). Data are analysed in a data-secured environment of Statistics Netherlands, facilitating that health can be studied prospectively over a lifetime across data sources.

In summary, with ELAN, the collaborating regional healthcare organizations aim to facilitate (epidemiological) research on many different topics (e.g. etiological, prediction, diagnostic, descriptive, syndemics, healthcare costs, disease trends) in order to help improve integrated patient care, health outcomes, cost-effective person-centred healthcare and alignment of health policies across care settings.^3^

## Data collected

The number of individuals in the data infrastructure is increasing. At present, ELAN GP contains information on 734 000 individuals, but it is anticipated to surpass >1 million participants in 2024. ELAN contains data from regional routine healthcare from GPs, hospitals, mental healthcare organizations on medical information (e.g. diagnoses, medical history, laboratory findings, measurements, medication prescriptions, referral information) and information on pregnancies, births and first 28 days of newborn children (Perined).^15^ Medical information is combined with data from national data sources and municipal registries, with, among others, information on demographics, mortality, health expenditure, COVID-19, social determinants of health (SDOH; income, education, ethnicity by country of origin, urbanization), claims-based diagnoses (mental healthcare, hospital and chronic care) and municipal information on the utilization of the social support act (broad categories; see Table 3). Data were collected from 2007 onwards, with an ongoing annual update. Via a secure link, data are uploaded to Statistics Netherlands, which functions as the trusted third party, meaning that this organization pseudonymizes the data and attaches a unique number to individuals, which allows individual linkage across the different sources. Harmonizing the data happens at various stages in the process. Data are uploaded by the (healthcare) organizations to the data safe at Statistics Netherlands. Different health organizations with different information systems may deliver data in different formats and with different structures. These data are stored as is, and harmonization takes place when data sets for the various research projects are compiled by data managers.

**Table 3.** Measurements available in the Extramural Leiden University Medical Center Academic Network

Source	Measurements
GPs and hospitals	Laboratory findings and measurements (e.g. glucoses, creatinine, haemoglobin, cholesterol and lipids)
	Information in structured fields (e.g. smoking status, blood pressure, oxygen level, alcohol use)
	Diagnoses (ICPC/ICD-10/DBC)
	Referral information
	Medication (ATC8)
	Contraindication for medications (ATC8)
	Contact information (e.g. frequencies, GP, emergency room visits, in- and outpatients)
	Procedures [diagnoses–treatment combinations (DBC), hospital care]
	Custom (e.g. results calcium score, text-mining smoking information)
SN	Demographic information (e.g. country of origin, marital status, number of household members)
	DBC, hospital care
	Healthcare costs (long-term care act and health insurance act; e.g. GP, hospital, physiotherapy, specialized psychiatric care)
	Medication (ATC4)
	National cause-of-death registry (ICD-10)
	Socio-economic information (e.g. household/personal income, debts, highest attained education, occupation, socio-economic area code)
	Health monitor (survey, random population samples 2012, 2016, 2021)
	Other (youth disability aid, parenting support, children’s psychiatric care 2016 and onward)
	Environment (sewage measurements, air pollution)
Municipal health service	COVID-19
Municipal social support	Disability tools and support in the domain of the Social Support Act (2017–present)
Open source	Neighbourhood quality-of-life ranking meter (every other year), criminality (2010–present)
Psychiatry	Diagnoses (DSM 5/DBC), GAF scores
Perined	Information on pregnancies and births from gynaecologists and midwives (e.g. hypertensive disorders of pregnancy, gestational diabetes, birthweight)

ATC, Anatomical Therapeutic Chemical code; DBC, diagnosis–treatment combination; DSM, Diagnostic and Statistical Manual of Mental Disorders; GAF, Global Assessment of Functioning; GP, general practice; ICD-10, International Classification of Diseases 10th Revision; ICPC, International Classification of Primary Care; SN, Statistics Netherlands.

In an online repository, code for ingestion, cleaning, imputation and pre-processing is standardized and available to the public. Code for common analysis task is also gradually added to this repository, as will code for the complete reproduction of results presented in future publications based on ELAN data. All analyses are done within the secured data environment of Statistics Netherlands and, after analyses, Statistics Netherlands performs an output check; this check certifies that data are non-retraceable to individuals (anonymized).

### Updates and known information on missing data

- Information on Statistics Netherlands and from regional routine healthcare data from GPs, hospitals, Perined and mental healthcare are annually updated.
- For new data sources, when available, data from 2007 onwards are retrospectively included.
- When individuals move out of the area, information on important outcome measures such as medical diagnoses and mortality are available in national registries (medical diagnoses from a declaration-based register and national cause-of-death register). Approximately 1% of the Dutch population emigrates, with no follow-up information on diagnoses and deaths.^16^
- When Dutch citizens die in another country, the cause of death remains unknown (registered by the National death national data sources as ‘unknown cause of death’, ICD10 R99); in 2018, this was 1.2% of all deaths.^16^
- Known information on missing data in GP files is described in Table 4.

**Table 4.** Percentages of missing data in the general practice total population and subgroups in the Extramural Leiden University Medical Center Academic Network

			**Age (years)** ^b^		
Characteristic	Overall GP	**Diabetes** ^a^	<18	18–65	≥65
*N*	734 519	49 689	238 282	422 807	73 430
Demographics (%)^c^					
Sex	0	0	0	0	0
Age	0	0	0	0	0
Person-years	0	0	0	0	0
Disposable household income	4.5	1.6	6.7	3.5	3.2
Education	34.4	64.4	4.4	40.9	94.0
Country of origin	0	0	0	0	0
Municipality	0	0	0	0	0
Laboratory findings and measurements (%)^c^					
ALAT	56.0	16.7	78.4	47.6	32.1
Creatinine	49.7	6.4	76.0	39.6	23.0
Haemoglobin	43.1	11.4	62.1	35.7	24.1
Sedimentation rate	48.6	16.4	68.8	41.0	26.7
Total cholesterol	64.3	9.8	95.7	51.7	34.9
TSH	51.6	20.3	72.3	43.2	32.9
Smoking status	69.3	17.1	91.6	60.0	50.2
Systolic blood pressure	54.5	7.6	81.7	44.1	26.2
Weight	64.9	12.3	75.0	62.6	44.6

ALAT, alanine aminotransferase; GP, general practice; TSH, thyroid-stimulating hormone.

a Population with a type 2 diabetes diagnosis before or during follow-up.

b Age when entering the data infrastructure (entry date before 2007; date was capped at 1 January 2007).

c Percentage of missing data.

## Data resource use

**Figure 2.**
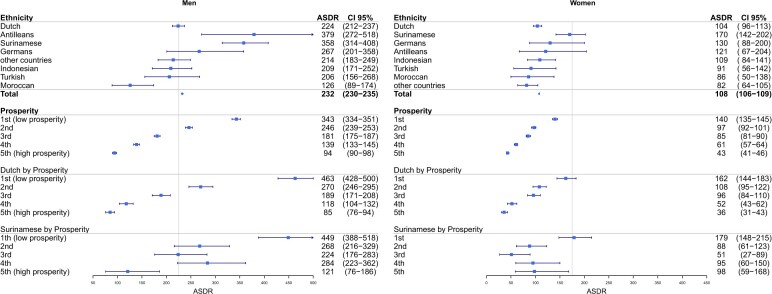
Age-standardized cardiovascular death rates in the city of The Hague, the Netherlands, reproduced from Kist *et al*.^17^ ASDR, age-standardized cardiovascular death rate (/100 000 person-years), standardization to the World population, 2007–18 (with 95% CI). Cut-off line, European Society of Cardiology-determined ASDR cut-off for high- and low-risk countries, cut-off men 225/100 000, women 175/100 000. Cardiovascular deaths, International Classification of Diseases 10th Revision diagnoses I00–I99. Ethnicity, according to country of birth (person or parent). Prosperity, disposable household income combined with household wealth, in quintiles of the population, 2012–18

The ELAN data infrastructure has been used for a wide range of research projects. A list of publications can be found on the ELAN research website (www.elanresearch.nl). Research projects showed large health disparities in cardiovascular death and morbidity in ethnic and socio-economic subgroups, with the number of cardiovascular deaths 5 times higher in low socio-economic groups compared with high socio-economic subgroups, and a 2–3.5 times higher occurrence of cardiovascular death in Surinamese (mainly South Asian) and Caribbeans (Figure 2).^17^ In the external validation of the primary care prediction models (SCORE2 and SCORE2-DM), cardiovascular events were underpredicted in Surinamese-South Asians and low socio-economic subgroups.^18^ In a multi-ethnic diabetes study, a non-Western origin was positively associated with the development of diabetes complications.^19^ Another study performed with ELAN data found that people with diabetes with and without a registered mental disorder showed no difference in the achievement of diabetes treatment targets.^20^ Two descriptive studies on antibiotics use showed a lack of registration of side effects and lower prescription of antibiotics during the SARS-CoV-2 wave compared with the 2020 influenza season.^21^^,^^22^ Another two studies explored the relationship between persistent somatic symptoms and symptoms, medication use and referrals.^23^^,^^24^ A study explored the predictors of inappropriate proton pump inhibitor use.^25^ There was a natural language-processing study on lifestyle characteristics.^26^ Also, choices made in pre-processing (Dutch) routine healthcare data for research were described.^27^

Ongoing research projects focus on a large variety of topics, namely cardiometabolic, SDOH, mental healthcare, surgery, orthopaedics, infectious diseases, antibiotic use, lifestyle interventions, neighbourhood interventions, pollution and diseases, kidney diseases, sex, age and gender differences, ageing population, syndemics in vulnerable populations, mental health disorders and cognitive impairment. Other projects concentrate on developing a transmural benchmark, tracking disease occurrences, monitoring disease trends and conducting implementation research to assess the impact of population health management and prevention projects. Current international collaborative efforts with similar data infrastructures in Australia, Scotland and Denmark will allow cross-border comparisons on diabetes care and health outcomes.

## Strengths and weaknesses

ELAN has various strengths. First, traditional cohorts usually have an underrepresentation of individuals from a low socio-economic background and/or with different ethnicities, whereas routine healthcare databases and registries are inclusive of all individuals in countries with a universal healthcare system (as the Netherlands is). Second, the combination of multilevel routine healthcare data from GPs, hospitals and mental healthcare, linked with the wealth of information on Statistics Netherlands, is unique, making ELAN the only data infrastructure so far in the Netherlands in which detailed information from these sources is combined for research on health inequities/disparities and other topics. Last, in order to follow trends after healthcare changes, next to information on determinants of health, healthcare costs are also available in the data set (albeit currently with a delay of 2–3 years).

This data infrastructure also has several limitations. First, routine healthcare data are recorded for patient care, not for research. Research biases can partly be mitigated by combining the variables and outcomes from different sources longitudinally. Second, in routine healthcare data, information on variables, such as smoking, cholesterol and blood pressure, is often missing. Missingness is comparable with those of other established observational routine healthcare data infrastructures such as QResearch Practices and Clinical Practice Research Datalink, and work is being done on improving data quality through complementing coded data using free text analytical techniques (text mining and natural language processing).^28–30^ Finally, the country of origin is the only available source as a proxy for ethnicity. Information on self-perceived ethnicity is not available in the data set.

## Data resource access

ELAN is governed by the ELAN LUMC Management Team. Requests for collaborations by national and international academic parties are welcomed on broad topics and can be addressed to ELAN at the Leiden University Medical Centre (www.elanresearch.nl or D.O.Mook-Kanamori@LUMC.nl).

Research proposals are assessed on scientific quality; public benefit; medical ethical considerations; adherence to Dutch legislation, General Data Protection Regulation, Statistics Netherlands legislation and risk for study patients, according to the ELAN Scientific Research Process.

The data underlying this article cannot be shared publicly due to Dutch legislation to ensure the privacy of individuals*.* For approved collaborative projects, data will be shared.

## Ethics approval

ELAN adheres to Dutch and European privacy and research legislation, and procedures have been implemented to protect patient confidentiality. Routinely collected data were anonymized through a trusted third party and Statistics Netherlands to prevent identification of individuals by researchers. In accordance with Dutch legislation, GPs and hospitals informed individuals about the use of their anonymized data for research purposes and individuals could withdraw via an informed opt-out procedure; informed consent from individuals in the study was waived and not obtained. For this waiver, the appropriate approval that the study is not subject to the Medical Examination Act was granted, after evaluation of the research protocol by the authority of the area (Medical Ethical Committee LUMC Leiden, under reference number G18.070).

## Supplementary Material

dyae099_Supplementary_Data
