# Supplementary material for: Data Resource Profile: Extramural Leiden University Medical Center Academic Network (ELAN)
Source: Int J Epidemiol. 2024 Jul 24;53(4):dyae099. doi: 10.1093/ije/dyae099 (PMC11269676; doi:10.1093/ije/dyae099)
Supplement: dyae099_Supplementary_Data [file dyae099_supplementary_data.docx]

**Supplementary tables**

**Supplementary Table S1, Characteristics of the general practitioner patient population of the Extramural Leiden University Medical Center Academic Network (ELAN), years 2007-2022**

**Legend**

ELAN**,** Extramural Leiden University Medical Center Academic Network

GP, data on the citizens of the ELAN area from the general practitioner’s/primary care population

SD, standard deviation

IQR, Interquartile range

Urbanisation, defined by number of addresses per square kilometre

Disposable household income, quintile boundaries by the quintile distribution of the Netherlands

Educational level, highest attained and followed educational level

Country of origin, by country of origin of individual or one/both parents (9 largest subgroups of the area)

**Supplementary Table S2, Grouped main causes of death according to the International Classification of Diseases, 10^th^ revision (ICD10)**

| **Causes of death** | **ICD10 codes** |  |
| --- | --- | --- |
| Certain infectious and parasitic diseases | A00–B99 |  |
| Neoplasms | C00–D48 |  |
| Diseases of the blood and blood-forming organs | D50–D89 |  |
| Endocrine, nutritional and metabolic diseases | E00–E90 |  |
| Mental and behavioural disorders | F00–F99 |  |
| Diseases of the nervous system | G00–G99 |  |
| Diseases of the circulatory system | I00–I99 |  |
| Diseases of the respiratory system | J00–J99 |  |
| Diseases of the digestive system | K00–K93 |  |
| Diseases of the skin and subcutaneous tissue | L00–L99 |  |
| Diseases of the musculoskeletal system and connective tissue | M00–M99 |  |
| Diseases of the genitourinary system | N00–N99 |  |
| Pregnancy, childbirth and the puerperium | O00–O99 |  |
| Certain conditions originating in the perinatal period | P00–P96 |  |
| Congenital malformations and chromosomal abnormalities | Q00–Q99 |  |
| Diseases not elsewhere classified | R00–R99 |  |
| External causes of morbidity and mortality | S00–T98 | V01–Y98 |

ICD10 codes, International Classification of Diseases, 10^th^ revision coded causes of death
